# Radiation safety issues regarding the cremation of the body of an I‐125 prostate implant patient

**DOI:** 10.1120/jacmp.v2i3.2611

**Published:** 2001-09-01

**Authors:** William Que

**Affiliations:** ^1^ MPCS Ryerson University 350 Victoria Street Toronto Ontario Canada; ^2^ M5B 2K3 and Department of Medical Physics Toronto Sunnybrook Regional Cancer Center 2075 Bayview Avenue Toronto Ontario Canada M4N 3M5

**Keywords:** cremation, I‐125, prostate implant

## Abstract

Radiation exposure to the public is estimated if the body of an I‐125 prostate implant patient is cremated. Precautions regarding the handling of cremated remains are suggested. Cremation can be performed safely at any time.

PACS number(s): 87.52.–g, 87.53.Jw

## I. INTRODUCTION: IS CREMATION ALLOWED?

In the past few years, prostate implant using radioactive I‐125 seeds has become a very popular treatment option for men with prostate cancer. These patients have a good prognosis and are expected to be long term survivors. Since the I‐125 half life is about 60 days, the probability of a prostate implant patient dying with a significant amount of radioactivity in the body is very low. At Toronto Sunnybrook Regional Cancer Center, we have performed about 250 prostate implants in 3.5 years, and all patients are still alive. According to the experience at Toronto Sunnybrook Regional Cancer Center, the average total implanted apparent activity for an I‐125 prostate implant is about 35 mCi. The total activity inside the seeds is about 1.6 times larger, or about 60 mCi. Twenty months (10 half‐lives) after the implant, the amount of radioactivity in the body will have reduced to only 60 *μ*Ci. Therefore, cremation of bodies with significant amounts of radioactive I‐125 can be treated as isolated incidents. Such incidents can occur when the patient dies from a motor vehicle accident or heart attack, and institutions need to set up guidelines to deal with the issue of cremation. In this article, we present an analysis on the possible dose to the public if the body is cremated, and suggest some precautions regarding the handling of cremated remains.

Very little in the published literature discusses the cremation issue. NCRP37,^1^ in principle, allows the cremation of cadavers containing radioactive isotopes, but it is a 31 year old document and is under revision. When we looked into this issue, we contacted many sources and got conflicting recommendations. A health physicist told us bluntly that the body of a prostate implant patient cannot be cremated. On the other hand, since AAPM TG56^2^ recommends that unwanted I‐125 seeds can be discarded into regular garbage after storing for 10 half‐lives (or 20 months), the implication is that the question is not whether cremation is allowed, but when cremation is allowed. A Canadian provincial cancer agency recommends that the body of an I‐125 prostate implant patient not be cremated within two years from the date of the implant. We contacted several AAPM task group members and AAPM representatives to the U.S. Nuclear Regulatory Commission (NRC), but received no clear answers. One AAPM representative told us that when a patient treated by his institution died, the prostate was removed from the body before the body was cremated. Other medical physicists told us of cases where cremation was performed with the seeds still in the body. This conflicting information created the motivation for writing this article, to clarify the issues.

Some individuals assume that the regulations, which apply to the waste disposal by the licensees, are also applicable to the crematoria. This assumption is incorrect. After some considerable efforts, we found two cases of cremation of the bodies of I‐125 prostate implant patients documented on the U.S. Nuclear Regulatory Commission website (at http://www.nrc.gov/NRR/DAILY/000727mr.htm and http://www.nrc.gov/NRR/DAILY/000413mr.htm). In one case, a patient died in July 2000, only five days after an I‐125 prostate implant. The estimated activity in the body was 9.8 mCi. With the permission of NRC, the body was cremated with the seeds inside the body. A radiation survey was performed on the cremated remains, and an exposure rate of 2 mR/h was measured in contact with the plastic bag containing the cremated remains. The radiation level dropped to background level when the plastic bag was placed in a metal urn. The second case involved the cremation of a body with 12 mCi of I‐125, but fewer details were given.

Although these two documented cases indicate that cremation is allowed, we feel there is still a need to present an analysis to provide an understanding of the issue. Furthermore, there are radiation safety issues related to the handling of the cremated remains, and these issues have not been documented previously. We believe that the publication of this article will be useful for radiation safety officers in clinical institutions performing I‐125 prostate implants.

## II. THE CREMATION PROCESS AND LIKELY DOSE TO THE PUBLIC

According to the Cremation Association of North America (CANA),^3^ cremation generally takes 2–3 hours in a sealed cremation chamber, during which time the temperature ranges between 1400–2100 °F (760–1149 °C), and the airflow rate is 2000–2500 cfm (cubic feet per minute). It is followed by a cooling period of 1 hour, during which the airflow rate is 500–1000 cfm. The product of this process is three to nine pounds of cremated remains or bone fragments. Some crematoria process cremated remains to reduce the space required for them, while other crematoria do not alter the condition of cremated remains. Cremated remains are placed in an urn and are either stored or buried, but some families may choose to scatter the cremated remains. Using midrange values, we find that the air volume released during the 2.5 hours of cremation is $3.4\times 10^{5}$ cf or 9585 $m^{3}$, and the air volume released during the cooling period of 1 hour is $4.5\times 10^{4}$ cf or $1274m^{3}$, making the total air volume released per body to be 11000 $m^{3}$.

From the case report of July 2000, mentioned earlier, one can infer that most of the I‐125 was released with the air, through the stack, due to the seeds rupturing during cremation. According to the exposure rate published in Table 1 of Ref. 4, the exposure rate of an unshielded point source of 10 mCi should be about 200 mR/h at a distance of 8 cm, or about 100 times the value mentioned in the case report. Likely, only a few percent of the radioactive material was left in the cremated remains.

In order to estimate the dose to the public due to the release of I‐125 through the stack, some assumptions have to be made and a model for calculation needs to be used. We use the Gaussian plume model, adopted in NCRP123,^5^ to calculate the I‐125 concentration *C* at a horizontal distance *x* (in m) from the stack. In this model,

(1) $$C=fQ\;\exp[-0.5{\left(H/\sigma_{z}\right)}^{2}]/(\pi u\sigma_{y}\sigma_{z}),$$

where *f* is the fraction of time the wind blows toward a receptor, *Q* is the effluent release rate at the stack, *H* is the stack height from ground level, *u* is the mean wind speed, ${\sigma_{z}=0.06x/(1+0.0015x)}^{½}$, and ${\sigma_{y}=0.08x/(1+0.0001x)}^{½}$. In our calculation, we use f=1, while for *u*, we use the recommended value 5 of 2 m/s. We also assume that: (a) the body to be cremated contains 60 mCi of I‐125, representing the scenario that the patient died right after the implant; (b) all of the amount of I‐125 is released through the stack at a steady rate in 3 hours, resulting in an effluent release rate of Q=5.6μCi/s; (c) the stack height of the crematorium is 10 m above ground level. The 10 m stack height corresponds to the value used by the U.S. Environmental Protection Agency's Office of Pollution Prevention and Toxics in modeling effects of pollution. Higher stacks will result in less dose to the public. Note that some states require a minimum stack height of 12.2 m (40 feet).

The model in Eq. (1) predicts low concentration *C* at both small and large distances, with a maximum *C* at an intermediate distance *x*. For the above parameters, we find the maximum concentration of I‐125 to be at a distance of about 130 m from the stack, with $C=4.5\times 10^{-3}\mu {\text{Ci}/m}^{3}$. Note that the I‐125 concentration at the stack is about 6 *μ*Ci/m^3^, hence there is a dilution of more than 1000 times. Since the volume of air breathed by the “Reference Man” doing “light work” is 2×10 ^4^ ml/min or $0.02m^{3}/\text{min}$,^6^ at a concentration of $C=4.5\times 10^{-3}\mu {\text{Ci}/m}^{3}$, in 3 hours the “Reference Man” could inhale 0.03 *μ*Ci of I‐125. According to Table 1 of Ref. 6, an annual intake of 200 *μ*Ci of I‐125 through inhalation would translate to a committed effective dose equivalent of 50 mSv. This means that a person inhaling 0.03 *μ*Ci of I‐125 would receive an effective dose equivalent of 0.0075 mSv, which is more than two orders of magnitude less than 1 mSv, the annual radiation dose limit for the public.

## III. PRECAUTIONS

A few percent of the radioactivity in the body is likely to be contained in the cremated remains. In the worst scenario, that the patient dies immediately after the implant, the residual radioactive material in the cremated remains would be in the range of 600–6000 *μ*Ci. Since inhaling 4 *μ*Ci, or oral ingestion of 2 *μ*Ci, of I‐125 translates to a radiation dose of 1 mSv (derived based on information in Ref. 6), the following precautions appear necessary when the body is cremated less than 10 half‐lives (or 20 months) from the date of the implant.

(A) The cremationist should wear a mask when handling cremated remains. We also recommend the use of rubber gloves during handling or washing hands afterwards. Although the cremationist is expected to always wear a mask when handling cremated remains, regardless if radioactive material is present, in reality, it is known that this rule is not followed strictly by workers. It is therefore necessary for the radiation safety officer of the clinic to give explicit instruction on this to the crematorium.

(B) Cremated remains should not be processed, and should be put in a metal urn for storage or burial. Processing the cremated remains could cause unnecessary contamination and exposure.

(C) In keeping with the recommendation of AAPM TG‐56, that unwanted seeds should decay in storage for 10 half‐lives before discarded into regular garbage, cremated remains should not be scattered until 10 half‐lives, or 20 months, from the date of the implant.

(D) It would be a good idea to send a physicist or delegate from the clinic to the crematorium to explain the precautions and perform a radiation survey immediately after cremation.

Finally, we note that sometimes the body is embalmed before cremation. Usually there is no need to make an incision on the pelvis of the body during embalming, but a large needle may be used to draw fluid from the body. Since I‐125 seeds are sealed sources, the body fluid should not be radioactive. According to Ref. 4, the average exposure rates around the patient immediately after an I‐125 prostate implant are 0.14 mR/h at 1 m in front of the abdomen, and 1.6 mR/h in contact with the abdomen. At such low rates, no special precautions are necessary for the worker performing embalming.

In conclusion, the body of an I‐125 prostate implant patient can be cremated safely at any time. If the body is to be cremated less than 20 months from the date of the I‐125 prostate implant, some precautions are needed in handling the cremated remains.

## ACKNOWLEDGMENTS

I would like to thank Peter O'Brien, Dale Water, and Donald Douthit for useful discussions.
